# Genotyping by Sequencing Revealed QTL Hotspots for Trichome-Based Plant Defense in *Gossypium hirsutum*

**DOI:** 10.3390/genes11040368

**Published:** 2020-03-28

**Authors:** Haris Ahmed, Mian Faisal Nazir, Zhoe Pan, Wenfang Gong, Muhammad Shahid Iqbal, Shoupu He, Xiongming Du

**Affiliations:** 1 Institute of Cotton Research, Chinese Academy of Agricultural Sciences, State Key Laboratory of Cotton Biology, Anyang, Henan 455000, China; ahmedharis25@gmail.com (H.A.); mfn121@hotmail.com (M.F.N.); panzhoe@caas.cn (Z.P.); shahidkooria@gmail.com (M.S.I.); heshoupu@caas.cn (S.H.); 2 Key Laboratory of Cultivation and Protection for Non-Wood Forest Trees, Ministry of Education, Central South University of Forestry and Technology, Changsha 410004, China; gwf018@hotmail.com; 3 Cotton Research Station, Ayub Agriculture Research Institute, Faisalabad 38000, Pakistan; 4 Zhengzhou Research Base, State Key Laboratory of Cotton Biology, Zhengzhou University, Zhengzhou 450001, China

**Keywords:** QTL mapping, Trichomes, genotyping by sequencing (GBS), pubescence, QTLs, insect pests

## Abstract

Cotton possesses certain physical features, including leaf and stem trichomes that help plants deter damage caused by insect pests, and to some extent, from abiotic factors as well. Among those features, trichomes (pubescence) hold a special place as a first line of defense and a managemental tool against sucking insect pests of cotton. Different insect pests of cotton (whiteflies, aphids, jassids, and boll weevil) severely damage the yield and quality of the crop. Likewise, whiteflies, aphids, jassids, and other insect pests are considered as potential carriers for cotton leaf curl viruses and other diseases. Genotyping by sequencing (GBS) study was conducted to understand and explore the genomic regions governing hairy (Pubescence) leaves and stem phenotypes. A total of 224 individuals developed from an intraspecific cross (densely haired cotton (Liaoyang duomao mian) × hairless cotton (Zong 128)) and characterized phenotypically for leaf and stem pubescence in different environments. Here we identify and report significant QTLs (quantitative trait loci) associated with leaf and stem pubescence, and the response of plant under pest (aphid) infestation. Further, we identified putative genes colocalized on chromosome A06 governing mechanism for trichome development and host–pest interaction. Our study provides a comprehensive insight into genetic architecture that can be employed to improve molecular marker-assisted breeding programs aimed at developing biotic (insect pests) resilient cotton cultivars.

## 1. Introduction

Trichomes (hairs) are present on the leaf and stem surface of many *Gossypium* species. The word hirsutum, derived from the Latin word “*hirsutus*” means hairy [1]. The economic importance of certain plants is axiomatic as they are used to feed and earn livelihoods. Cotton is mainly produced for its industrial use in the textile industry. Crop plants live in labile conditions and are more prone to certain environmental and biotic stresses. Among biotic factors, many species of insect pests, i.e., whiteflies, aphids, jassids, and boll weevil cause severe damage, affecting cotton yield and quality [2]. Insects are carriers for many diseases, such as cotton leaf curl virus that uses whitefly [3] as a carrier to spread the disease. To cope with the ever-increasing damage caused by insect pests, the cotton plant has evolved morphological features that protect it from the damage of sucking, and herbivores pests, such as leaf trichomes [4]. 

Cotton possess several special plant traits, i.e., trichomes, which have the potential to manage pest population. In general, these are specialized unicellular or multicellular protuberances from epidermal cells present on leaf and stem surfaces, act as the first line of defense that protect plants from insects, herbivores (by obstructing their movement), oviposition, excessive transpiration, low temperature, and UV radiation [5,6,7]. Based on their functions, trichomes can be classified into glandular (functions to secrete certain chemicals and secondary metabolites to protect plants from herbivores and pathogens) and non-glandular (functions to reduce heat loads, increase plant ability to tolerate freezing, help in seed dispersal, and protection of plant tissues from UV light) trichomes [8,9,10]. In the absence of trichomes, plants are more prone to damage caused by biotic and abiotic factors, conversely increasing the interdependence on pesticides [11]. Distribution and development of trichomes are temporally and spatially controlled, i.e., at early developmental stages trichomes are present just near the base of leaves, whereas they are found on both the adaxial and abaxial surfaces of the leaf at the adult vegetative stage [12]. The amount of hairiness in *Gossypium* species varies with the extent of susceptibility or resistance to sucking insect pests [2]. Trichomes are mainly composed of cellulose and other less important substances that are usually of low nutritional value; thus, insects gain less weight, and eventually, increased mortality [4]. The presence of glands on trichomes makes it more lethal, structurally and chemically, because it produces secondary metabolites that can deter herbivorous organisms [13]. 

Trichomes share some common developmental and patterning pathways with cotton lint fiber in their early stage of development, Although leaf trichomes and cotton lint fibers are different morphologically [14]. Cotton lint fiber, which is of main economic importance, is an unbranched and extremely long type of trichomes, and these are extensions of seed epidermal cells [15,16]. Close molecular phylogenetic relationships have been found between the *Gossypium* (Malvales) and Brassicales (*Arabidopsis*) species, which found that the regulatory mechanism controlling leaf trichomes initiation in *Arabidopsis* might also be present in cotton [17,18,19]. Trichome initiation and development are best understood and studied in *Arabidopsis;* few genes play a crucial role in trichome initiation, including *GLABROUS1* (*GL1*), *TRANSPARENT TESTA GLABRA1* (*TTG1*), and *GLABRUS3/ENHANCER OF GLABRA3 (GL3: EGL3)* [20], and at least 40 genes involved in trichomes developmental pathways [21].

In *Gossypium* species, previous reports suggested multiple QTL (quantitative trait loci) hotspots on chromosome A06 for leaf pubescence using BC1 and BC2 as a mapping population, employing randomly amplified polymorphic DNA (RAPD) markers [22,23]. In *Gossypium barbadense* L. and *Gossypium hirsutum* L. on chromosome six, a retrotransposon insertion was found in *homeodomain Leucine Zipper gene* (*HD1*) resulting in hairless stem phenotypes [1,24]. 

In the past, different approaches were used to underpin the genetic and molecular mechanisms underlying leaf and stem pubescence. The power of these approaches is limited and has been enhanced using next-generation technologies for the creation of genetic maps [25]. By using different platforms of next-generation sequencing (NGS) technology, we can now get full coverage of genomes through SNP (single nucleotide polymorphism) markers without any need for reference genome [26]. Advancements in NGS reduced the cost of DNA sequencing up to the point where genotyping by sequencing (GBS) can easily be used for more diverse and large genome species. Restriction enzymes (methylation-sensitive) used in GBS can efficiently outdo the complexity and redundancy of genomes [27]. This approach has been widely used for trait mapping and diversity studies in different crops like chickpea [28], cabbage [29], maize [30], wheat [31], rye [32], and common bean [33].

In the present study, we have used GBS approach for SNP calling and QTL mapping to identify genes underlying the major/stable QTLs, involved in leaf and stem trichome formation. We also identified QTLs and candidate genes that could be turned on during insect host interaction. To the best of our knowledge, this is the first study to identify QTLs and candidate genes for leaf and stem pubescence in cotton using the genotyping by sequencing (GBS) approach. The results of this study will be helpful to understand insect–host interaction and the development of pest tolerant cotton cultivars.

## 2. Materials and Methods 

### 2.1. Plant Material

The mapping population consisted of 224 F_2_ individuals derived from a cross between densely haired with hairless cotton (Liaoyang duomao mian (LDM) × Zong 128). The female parent LDM is known for its profuse hairy leaf and stem, while the male parent, Zong 128, does not possess leaf or stem hairs. Seeds taken from each individual of F_2_ bulked and divided into two parts for growing and evaluated for leaf and stem hairs, aphid rate, and aphid grade in Anyang research station, Henan province, China, and Sanya research station, Hainan, China. The experimental layout adopted for this study was the randomized complete block design (RCBD) with three replications. The plot size was a 5 m long row with 0.75 m of line spacing and 0.30 m of plant spacing.

### 2.2. Data Recording and Statistical Analysis

There were two leaf hair scoring methods, qualitative and quantitative [11], respectively. The former includes grading the leaf hairs from sparsely hairy, to medium, and profuse hairy, denoted by 1, 2, and 3, respectively, while the latter included trichomes count with the help of a microscope. In the present study, stem hairs were recorded as 1 (being no hairs), 2 (as medium), and 3 (as dense hairs). The trait stem pubescence amount (SPA) used to denote the amount of stem hairs on the surface of the stem and leaf pubescence amount (LPA) used to denote the amount of hairs on the surface of the leaf. The number of leaf hairs was counted with the help of a light microscope (Olympics 1 × 71). Five leaves per plant were selected for leaf hair count and data was recorded from the basal, mid, and apical areas of the leaf. For this, the lower surface of the leaf was placed under a microscope and the number of trichomes was counted inside a 6-mm ring, as described by Wright et al. [34]. Data was recorded from three different places of the leaf and then pooled to get the average, for subsequent statistical and genetic analysis. Aphid population was calculated by visual means and five plants per line were selected for aphid count, quantitatively as aphid rate and qualitatively as aphid grade. XLSTAT software was used to get means, standard deviation, and correlation values between the traits.

### 2.3. DNA Extraction and Genotyping by Sequencing

DNA was extracted from fresh leaf samples obtained from the field. There were 224 individuals and 10 leaf samples taken from each parent. The standard CTAB (Cetyl Trimethyl Ammonium Bromide) method was employed as described by Paterson et al. [35]. Total DNA was quantified by Nano Photometer^®^ spectrophotometer (IMPLEN, Westlake Village, CA, USA), with the absorbance ratio at 260 and 280 nm. The DNA samples (with an absorbance ratio of around 1.8) was considered pure. 

GBS protocol as described by Elshire et al. [27] was performed at Beijing Novogene Bioinformatics Technology Company, for GBS and next-generation sequencing, using the Illumina HiSeq PE150 sequencing platform. GBS libraries were constructed by digesting genomic DNA with restriction enzyme combination MseI + NlaIII. TASSEL 3.0 software was used for SNP calling using *Gossypium hirsutum* v1.1.fa as reference genome [36]. For alignment, Burrows wheeler Aligner was used with default settings. After custom filtering, alignments, and deleting the missing and distorted data, 5205 SNP markers were identified and retained for further analysis. 

### 2.4. Linkage Map Construction

JoinMap v4.1 [37] was used for linkage group construction and 26 linkage groups (LGs) were obtained. Each group was assigned and named to its corresponding chromosome using BLASTN search results. The anticipated segregation ratios, 1:1 for one way cross or 1:2:1 for an intercross, were screened and tested by employing chi-square test on all markers. To map distances of recombination frequencies, the Kosambi map function was used with a recombination frequency of 0.40 and logarithm of odds (LOD) score set at 2.5.

### 2.5. QTL Mapping and Gene Mining

QTL analysis was performed using WinQTL Cartographer (version 2.5) [38]. The parameter set to map the QTLs was composite interval mapping (CIM). In this method, certain values and functions were set to map QTLs with precision, i.e., in our study, we used the forward and backward regression model with 1 cM (centimorgan) walking speed, probability of input and output was 0.01, and window size at 10 cM. The LOD threshold value was calculated by manual permutation method with the value set at 11.5, which is equal to 2.5 LOD score. Trait QTL values below a 2.5 score were considered not fit for further selection of significant and consistent QTLs. The LR (left to right) values generated by the QTL cartographer were then used in Map Chart (v2.2) software for visualization of QTL locations on the chromosome, along with their marker locations. 

QTLs with a high percentage of phenotypic variance R2 and higher LODs with overlapping behavior were retained for further analysis. Start and end positions of the markers, physical, and genetic locations of the respected QTLs used, yielded a group of genes expected to influence plant morphology. The group of genes and their functions were studied to narrow the most robust genes influencing plant trichomes. Gene ontology was carried out from online sources https://cottonfgd.org/, https://www.arabidopsis.org/index.jsp, and Phytozome (https://phytozome.jgi.doe.gov).

### 2.6. Gene Expression Level Analysis (qRT-PCR)

Fresh leaf and stem samples were collected from one-month-old cotton plants and total RNA was extracted using RNAprep Pure Plant Kit (Tiangen). First-strand complementary DNA (cDNA) was generated from 1 μg total RNA from individual replications using a PrimerScript RT Reagent kit (Perfect Real Time, TaKaRa, Japan). Quantitative real-time RT-PCR was performed using SYBR^®^ Premix Ex TaqTM (Perfect Real Time, TaKaRa, Japan), according to the manufacturer’s instructions. For internal reference actin transcript was added in each reaction. The expression levels of genes were calculated using equation 2^−ΔΔCT^ where ΔΔCt = (Ct target − Ct actin) sample X − (Ct target − Ct actin). For every sample, three technical and biological repeats were used. The primers for qPCR were designed online through Primer-blast [39] tool available at the National Center for Biotechnology Information (NCBI) and further confirmed through Oligo7 software. The primers used in this study are listed in Supplementary Table S5.

### 2.7. Synteny Analysis

In earlier studies, researchers explained the transference of hairiness from *Gossypium raimondii* to *G. hirsutum* [40]. To test this phenomenon, and the relationship between the candidate genes of *G. hirsutum* with *Gossypium raimondii*, *G. arboreum,* and *G. barbadense* we performed synteny analysis using online resources, https://www.cottongen.org/synview/search. Alignment and phylogenetic analysis was performed in MEGA X software [41]. Further visualization and optimization of the phylogenetic tree was performed using FigTree v1.4.4.

## 3. Results

### 3.1. Correlation and Phenotypic Evaluation

**Figure 1:**
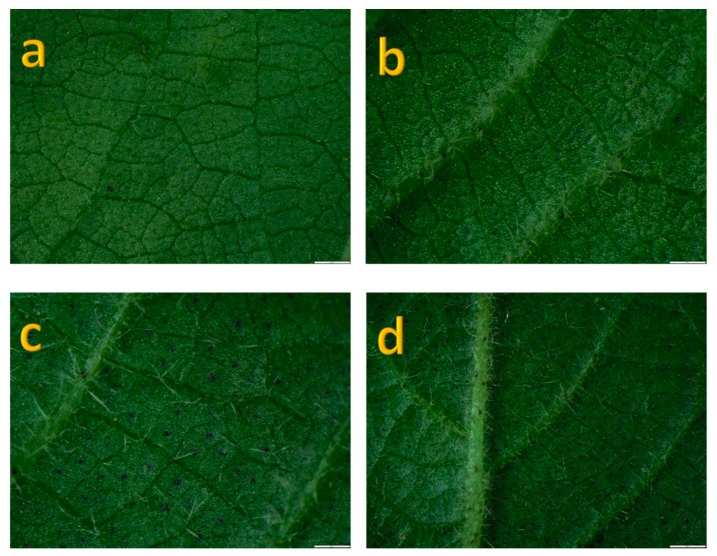
Microscopic images of leaf trichomes. A = no hairs, b = medium hairs, c and d = profuse hairy. Scale bars in (a–d) = 200 µm.

Summary statistics was performed to get mean, standard deviation, minimum, and maximum values (Table 1). Leaf pubescence amount (LPA) showed marked differentiation between two geographically distinct locations, Hainan Island (South China) and Anyang (Central China) (Table 1). Leaf pubescence amount described in Figure 1 as being no hairs to medium and profuse hairiness.

We found a significant positive correlation (Pearson correlation) (Table 2) between aphid rate and leaf and stem pubescence (0.235 and 0.365), which implies that an increase in the number of leaf and stem hairs will ultimately increase the pest population. A similar fashion was observed between aphid grade and leaf and stem pubescence (0.330 and 0.437).

### 3.2. Genetic Linkage Map for F2 Population

Initial screening for distorted and uninformative markers was performed to avoid over or underestimation of recombination frequencies that subsequently alter the genetic distances between the markers. A linkage map comprised of 26 linkage groups with 5205 SNP markers spanning a total of 7682.27 cM was constructed. Main features of the linkage map i.e., genetic distance, number of SNPs anchored on respected chromosomes, the average distance between markers, and the maximum gap between SNP markers are presented in Supplementary Table S1 and Supplementary Figure S1a,b,c. The minimum number of SNPs were found on D03 (21) while the maximum number of SNPs anchored on A06 (997). Likewise, the longest chain length was 1121.35 cM, maximum gap between the markers was 27.12 cM and the average genetic distance between the adjacent marker was 1.48 across 26 linkage groups.

### 3.3. QTL Mapping

**Figure 2:**
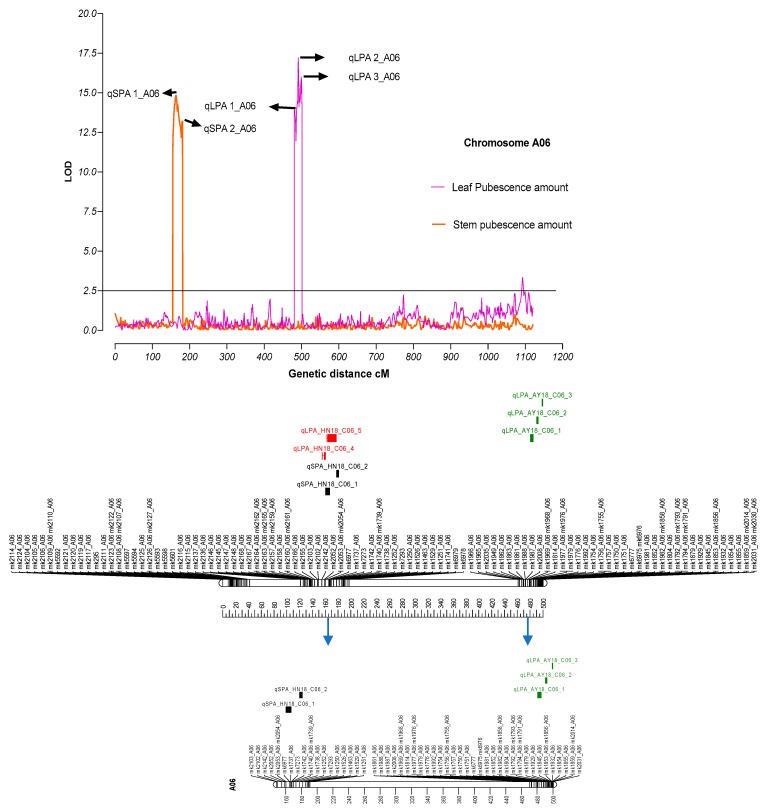
Physical representation of QTLs on chromosome A06 for leaf and stem pubescence. Vertical lines on the upper side of the figure indicating LOD values for leaf and stem pubescence against their genetic distances in centimorgan. Arrow signs show the location of QTLs for the respected trait. The lower portion indicates physical representation of QTLs on Chromosome A06.

Association was found between genetic linkage map (SNP information) and phenotypic data using WinQTL cartographer to estimate QTLs for traits under study. Only those QTLs with maximum LOD signal and highest phenotypic variance (R2) were retained for further analysis and discussion. We observed strong signals (LOD) on chromosome A06 for leaf (five QTLs; qLPA 1_A06, qLPA 2_A06, qLPA 3_A06, qLPA 4_A06, qLPA 5_A06) and stem pubescence (two QTLs; qSPA 1_A06 and qSPA 2_A06) (Figure 2, Table 3) (Supplementary Table S2). Genetic position of qSPA 1_A06 was 163.41 cM having a high LOD score of 14.85 with an observed phenotypic variance value 0.16. Another significant LOD signal (qSPA 2_A06) was observed at 180.71 cM for stem pubescence QTL with an LOD score of 13.22. The QTL, qLPA, 4 were found at 491.21cM, and under this QTL, we found the most informative genes were related to leaf pubescence (Table 4). Parental contribution for a specific trait towards the QTLs based on their additive values: seven QTLs were linked to a hairy phenotype-contributing parent (LDM) while only three QTLs were contributed by a hairless parent (Zong 128).

We also found three QTLs related to plant responses under aphid infestation. Two QTLs qAG 1,2 found on chromosome six at 266.01 and 274.01 cM (Supplementary Figure S2, Table 3) yielded interesting information, although their genetic position is different, but their physical location overlapped with stem pubescence QTLs. The physical co-localization of these two QTLs made it the most important QTL, because the overlapping QTLs will not only help maintain pubescence, but will also help initialize resistance mechanisms against insect pests.

### 3.4. Candidate Gene Identification

Exploration of genes underlying QTLs was carried out using genetic distance information and start and end position of the markers from the map file. There were around 300 genes identified after gene mining (https://cottonfgd.org/) (Supplementary Table S3). Gene sorting was carried out by identifying their role in regulating trichomes by the TAIR (The Arabidopsis Information Resource) online resource (https://www.arabidopsis.org) and previously published work. For stem and leaf pubescence (trichomes), we found 231 and 70 genes, respectively. Of these, very few are related to trichome formation or somehow involved in trichome-mediated responses by the plants. Interesting information was noted during gene mining that for some genes underlying stem pubescence, QTL overlapped with the genes for leaf pubescence. Out of 300 genes, only eight were found to have a role related to trichome formation or trichome-mediated responses (Table 4). Two previously reported genes *PER64* (Gh_A06G0019) and *TUBA3* (Gh_A06G0984) were directly involved in trichome formation. The former involved in molecular and biological functions, i.e., function in response to oxidative stress, metal ion binding, and heme binding. The *PER64* gene, reported in *Arabidopsis thaliana*, translated into a protein peroxidase that not only has the oxidative properties, but is also differentially expressed in cotton fibers that affect fiber elongation by participation in peroxidase catalytic pathways, microtubules synthesis, and ethylene mediated responses [42]. *TUA5* encodes a protein Tubulin alpha chain that involves molecular functions, such as GTPase activity, GTP binding, and structural constituent of cytoskeleton. This gene is also involved in the biological process, such as microtubule elongation, and most importantly, seed trichome elongation [43]. Both genes are involved in fiber development processes, although trichomes and fiber are morphologically different, but are related by their developmental and patterning pathways at the early stages of development. We identified 23 and 61 candidate genes (Supplementary Table S4, Table 4) underlying aphid grade and aphid rate QTLs (respectively) that might trigger response of the cotton plant under aphid attack. Trichomes are also related to production of secondary metabolites (such as gossypol and related compounds), have strong antifungal activity [44], and are considered as potential natural pesticides [45]. Glandular trichomes of cotton also involved in production of indirect defense responses mediated by herbivores induced plant volatile and play an important role to attract predators and parasitoids to herbivore infested plants [46]. The two QTLs identified for aphid rate on chromosome 17 and 22, harboring 61 genes. One gene, *TPR1* (Gh_D09G0835), was involved in the immune response of plants during pathogen infection [47] while another gene, *AGO5* (Gh_D09G0839), was involved in antiviral defense responses through RNA silencing [48]. Mineral nutrients are very crucial in plant defense responses, and among them, Zinc (Zn) plays a crucial role in plant responses to pests and diseases. We found a gene *ZAT5* (Gh_D09G0857) related to plant defense responses influenced by mineral nutrients [49]; this response is mediated by physical features of plants, i.e., trichomes, waxy cuticles, etc.

### 3.5. Phylogenetic Relationship of Hairy Genes

**Figure 3:**
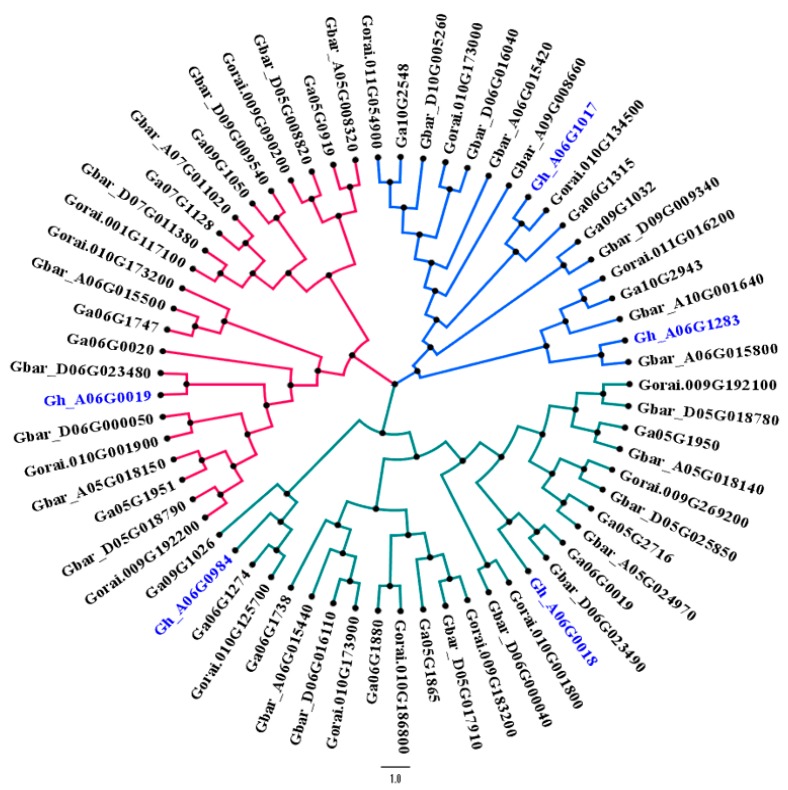
Phylogenetic relationship between candidate genes of *G. hirsutum* with *G. raimondii, G. arboreum,* and *G. barbadense.* The color scheme of the phylogenetic tree represents different clades. Gene IDs in the blue color are putative genes found in this study.

In previous studies, researchers explained the genetics of hairiness transferred from *G. raimondii* to upland cotton (*G. hirsutum*) [40]. To check this, we performed synteny analysis by using four candidate genes from *G. hirsutum* (which appeared to have some role in trichomes formation) TUA5 (Gh_A06G0984), CESA6 (Gh_A06G1017), PER64 (Gh_A06G0019), and GhEXPA1 (Gh_A06G0018), and 17, 15, and 26 genes from *Gossypium arboreum*, *G. raimondii,* and *G. barbadense,* respectively. Among three main clusters, a *G. hirsutum* gene CESA6 (Gh_A06G1017) co-localized with the *G. arboreum* gene, and was found to have a close relationship with the *G. raimondii* gene; similarly, other three candidate genes found in our study were in close relationship with *G. raimondii*, *G. arboreum,* and *G. barbadense* (Figure 3). An interesting fact to note from the dendrogram is that one of our candidate genes (Gh_A06G1283) is a homologue of the *G. barbadense* gene. Both genes are supposed to play a similar role due to their same evolutionary background, as revealed through synteny analysis.

**Figure 4:**
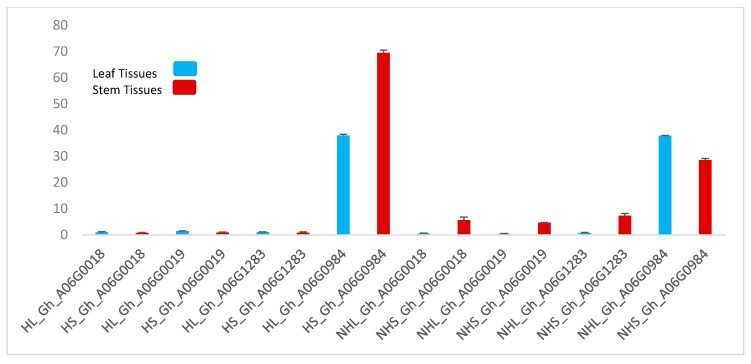
The gene expression profiles of four genes related to trichome formation. Two-color schemes showing leaf and stem tissues. HL: hairy leaf, HS: hairy stem, NHL: non-hairy leaf, NHS, non-hairy stem. Data represent the mean ± SD from three biological replicates.

To further validate the role of these genes in trichome formation, we performed qPCR. The results of the qPCR are shown graphically in Figure 4. Stem tissue TUA5 (Gh_A06G0984) showed elevated expression levels; this gene also showed a marked expression level in the hairy leaf as well. Although this gene showed the same expression level in the non-hairy leaf, less expression was observed in non-hairy stem tissue. 

## 4. Discussion

It is necessary to produce plant types that can withstand direct and indirect losses caused by insect pests. In the past, different approaches have been used to investigate the genetic mechanisms involved in trichome initiation and formation. Restriction fragment length polymorphism (RFLF) and RAPD (GLG-06_975_ linked with t_1_ locus) markers were used in the *Gossypium* species [23,34] to map genes for trichome formation; they found trichome related genes on chromosome six and named that locus as t_1_. A combined linkage map of RFLP–SSR–AFLP markers revealed nine QTLs related to hairiness, and identified a significant QTL localized on chromosome six [22]. The above-mentioned studies are in accordance with our findings that the QTLs controlling hairiness are present on chromosome six.

The paucity, relatively low level of DNA polymorphism, and incapacity of RFLP, AFLP, RAPD, SSR and other markers are some possible drawbacks to map QTLs/genes with certainty. Such limitations of markers make it unrealistic to study complex traits compared to recent available next-generation sequencing platforms. Genotyping by sequencing was used in this study to map QTLs related to leaf and stem pubescence because GBS is a cost-effective next-generation sequencing-based approach, and very accurate for high-density marker development [50]. GBS markers possess several advantages over other conventional and non-conventional markers, i.e., generic sample preparation, streamlined and easily available bioinformatics tools to work on genotyping goals (independent of reference genome), and most importantly, reduced representation of genome (redundancy) by employing restriction enzymes [51]. The most striking and extraordinary feature of GBS is the ability to genotype any population irrespective of the availability of parental data and to score SNP markers co-dominantly in a segregating population [51].

In the present study, we utilized GBS markers to map the QTLs and subsequently candidate genes for trichome formation. We developed a segregating population of 224 individuals by intraspecific cross of hairy white cotton with hairless cotton (Zong 128 x Liaoyang duomao mian (LDM)). We found 16 QTLs related to stem and leaf pubescence and 12 QTLs related to aphid population/plant response against aphids. Seven QTLs (for leaf and stem pubescence) and three QTLs (for aphid population/plant response against aphids) qualified for further analysis and gene mining. 

Phylogenetic analysis revealed a relatively close relationship between cotton and model plant *Arabidopsis thaliana* [17,18,19] and it may be inferred that genes for trichome formation in *Arabidopsis* can play a key role in cotton as well. Under stem pubescence QTL (qSPA 1_A06) we found a gene, *UPL5* (Gh_A06G1357), involved in the biological processes in *Arabidopsis* and showed response to jasmonic acid [52], which is a phytohormone that plays a significant role in trichome development in *Arabidopsis* [53]. Other genes underlying stem pubescence QTLs are *ALDH7B4* (Gh_A06G1257), which plays a role in heat stress and water deprivation conditions [54] and *MPE3* (Gh_A06G1253), which is involved in biological processes (GO:0042545) and plays a role in cell wall modifications. As the presence of trichomes also enable plants to withstand abiotic stresses (e.g., heat and drought stress), so these genes, *ALDH7B4 and MPE3,* can be attributed to trichome-mediated responses. 

We found four genes that may be involved in trichome development or initiation. The first gene is *TUA5* (Gh_A06G0984), which encodes a protein Tubulin alpha chain that plays a key role in polarized growth of trichomes on the surface of cotton ovules [43]. As discussed earlier, although leaf trichomes and cotton lint fibers are slightly different morphologically, they share common developmental processes [14], so we propose that this gene may play a key role in trichome development. Another gene, *CESA6* (Gh_A06G1017), involved in molecular as well as biological processes, i.e., plays a key role in cellulose biosynthetic process, cell wall organization, and multidimensional cell growth [55]. All of these processes lead to the formation of cellulose, which is not only the integral part of the cell wall but also a basic building block of trichomes. So, it may be inferred from here that this gene could be very important to manipulate and use for trichome augmentations. *PER64* (Gh_A06G0019), along with other genes, affect the fiber elongation process by partaking in ethylene response, microtubule synthesis, and peroxidase catalytic pathway. Consequently, the role of this gene is quite remarkable considering its contribution to fiber developmental processes. It can have a similar role for trichome formation on the surface of the leaf and stem. A homeodomain transcription factor *GhHOX3* proved to have a crucial role in the development of seed trichomes by the involvement of other cell wall loosening protein genes *GhRDL1* and *GhEXPA1*. This *GhEXPA1* (Gh_A06G0018) gene found in our study appears to have a role in cell wall expansion and combines with *GhHOX3* and *GhRDL1*, and promotes fiber elongation [56]. While working on *Gossypium barbadense,* Ding et al., (2015) found a *homeodomain leucine zipper gene (HD1)* that controls the hairiness mapped to chromosome A06. We also found a homologue of *HD1* gene (Gh_A06G1283) in our population on chromosome six underlying qLPA 1_A06 QTL, which is also a *homeodomain leucine zipper gene* (*HD-1A*), related with trichomes formation [24]. So, this candidate gene (Gh_A06G1283) could play a very important role in trichomes formation. The functional study of this gene could provide comprehensive insight into the mechanisms underlying leaf and stem pubescence and can be used for further gene manipulations. 

Apart from obstructing the movement of pests on the leaf and stem surface, trichomes aid and abet cotton plants to detect physical presence of insects and respond accordingly. We found four genes under aphid rate QTLs, directly related to plant response against pathogens. First, gene *TPR1* (Gh_D09G0835) located on the D09 chromosome—overexpression of this gene leads to the activation of plant defense responses. Under insect attack, this gene suppresses the negative regulators of immunity, which activates the resistance (R) protein-mediated immune responses [47]. The other three genes that we found in our study *AGO5* (Gh_D09G0839) [48], *ZAT5* (Gh_D09G0857) [57], and *GLO4* (Gh_A06G1246) [47] are also involved in plant responses towards pest attack. 

Under stem pubescence (qSPA 2) QTL, we mined a gene *TUA5* (Gh_A06G0984) that controls the formation of trichomes on cotton ovule. We postulated that this gene could play a key role in trichome formation; to check this hypothesis we performed qRT-PCR and found a high expression level of this gene in hairy stem tissues. The expression of this gene was also found a little elevated in non-hairy leaf and stem tissues, but when compared to hairy stem tissues, the expression was even higher. These results suggested that Gh_A06G0984 could most likely be the candidate gene under stem pubescence QTL that controls the trichome formation on chromosome A06. Further validation and exploitation are needed to develop a full understanding of the mechanisms involved in trichomes and trichome-mediated responses by cotton.

We narrowed down the QTL regions by using the gene function information in *Arabidopsis*, phylogenetic analysis and gene expression profiles. QTL under study contains plenty of genes that need to be narrowed down in an order to find the most suitable candidate genes, but this is laborious and time consuming. To prioritize candidate genes underlying QTLs, a computational approach based on algorithms would be very helpful. In a study, researchers developed QTG-Finder, a machine learning based algorithm to prioritize candidate genes by ranking them within QTL region [58]. These studies are useful to narrow down candidate genes and such algorithms should be implemented in studies aiming to predict causal QTLs and genes for traits of interest.

Our study provides comprehensive insight into QTLs/candidate genes underlying leaf and stem pubescence. The adversities caused by insect pests of cotton and other abiotic stresses are quite high, and development of cotton cultivars that can withstand and give sustainable yield under stress is of prime importance. We noted an interesting observation in our study that stem pubescence QTL and aphid response QTL shared overlapping physical positions. These QTLs/candidate genes may be involved in pleiotropic effects, i.e., similar QTLs/candidate genes partaking trichome formation as well as triggering plant hormones/chemicals in response to pest infestation. This point also strengthens the importance and reliability of QTLs/candidate genes under study. Functional studies of these genes will not only be helpful to understand their regulatory pathways and factors affecting trichomes, but can also be used in breeding programs aimed at producing pest resilient cotton cultivars.

## Figures and Tables

**Table 1:** Summary statistics of aphid population and trichomes count at two locations.

Variable	Minimum	Maximum	Mean	Std. Deviation
LPA_HN	0.330	40.670	13.230	10.040
LPA_AY	0.067	33.533	10.868	7.512
AR_AY	6.250	100.000	41.639	22.033
SPA_HN	1.000	3.000	2.099	0.634
AG_AY	1.000	5.000	2.746	1.577

LPA: leaf pubescence amount, AR: Aphid rate, SPA: Stem pubescence amount, AG: Aphid grade (number of aphids observed physically), HN: Hainan, AY: Anyang, number of samples: n = 224. Unit of measure = counting for leaf, stem pubescence, and for aphid infestation.

**Table 2:** Correlations between leaf and stem pubescence among 224 individuals with aphid population across two locations.

Variables	LPA_HN	SPA_HN	AR_AY	AG_AY	LPA_AY
LPA_HN	1				
SPA_HN	** 0.452	1			
AR_AY	* 0.235	** 0.365	1		
AG_AY	** 0.330	** 0.437	** 0.693	1	
LPA_AY	** 0.424	** 0.588	** 0.375	** 0.434	1

LPA: leaf pubescence amount, AR: Aphid rate, SPA: Stem pubescence amount, AG: Aphid grade (number of aphids observed physically), HN: Hainan, AY: Anyang. *indicate values are significant at alpha = 0.05, ** indicates values are highly significant at alpha = 0.01, *n* = 224.

**Table 3:** List of QTLs (quantitative trait loci) related to leaf and stem pubescence, aphid rate and aphid grade.

QTLs	Chromosome	Position(cM)	Start bp	End bp	#Genes	LOD	R2	Additive_effect	Dominant_effect	DPE
qSPA 1_A06	A06	163.41	88454646	95426881	146	14.85	0.16	0.52	0.17	LDM
qSPA 2_A06	A06	180.71	45870860	63574389	85	13.22	0.19	0.51	0.08	LDM
qLPA 1_A06	A06	481.81	90237872	93497786	55	13.94	0.31	6.30	−0.44	LDM
qLPA 2_A06	A06	491.21	46092	185343	12	17.22	0.39	6.8784	−1.15	LDM
qLPA 3_A06	A06	499.41	91597800	91713664	2	16.00	0.33	6.7449	−0.43	LDM
qLPA 4_A06	A06	158.81	95396635	95426819	1	6.45	0.05	5.19	2.95	LDM
qLPA 5_A06	A06	169.71	88651703	88804649	0	9.17	0.08	6.24	3.15	LDM
qAR 1_D09	D09	168.31	32855135	34029175	60	3.20	0.10	−7.3756	6.20	Zong 128
qAG 1_A06	A06	266.01	89207941	89941409	11	2.97	0.09	−0.54	0.39	Zong 128
qAG 2_A06	A06	274.01	88955098	89801937	12	3.21	0.10	−0.50	0.56	Zong 128

SPA: stem pubescence amount, LPA: leaf pubescence amount, AR: Aphid rate, AG: Aphid grade, #: Number, LOD: Logarithm of odds, R2: Phenotypic variance, DPE: Direction of phenotypic explanation, cM: centimorgan, bp: base pair, q: used in nomenclature for QTLs.

**Table 4:** List of candidate genes related to plant trichomes and response of plant against pests (Aphid).

Trait name	QTL	ID	Gene	Description	Start (bp)	End (bp)
Stem pub.	qSPA 1	Gh_A06G1357	UPL5	E3 ubiquitin-protein ligase UPL5	95,039,259	95,042,363
Stem pub.	qSPA 1	Gh_A06G1244	UBC34	Ubiquitin-conjugating enzyme E2 34	89,677,173	89,679,975
Stem and leaf pub.	qSPA 1 and qLPA 1	Gh_A06G1257	ALDH7B4	Aldehyde dehydrogenase family 7 member B4	90,765,222	90,768,706
Stem pub.	qSPA 1	Gh_A06G1253	MPE3	Pectinesterase 3	90,103,159	90,105,870
Stem pub.	qSPA 2	Gh_A06G0984	TUBA5	Tubulin alpha-5 chain	46,567,039	46,569,490
Stem pub.	qSPA 2	Gh_A06G1017	CESA6	Cellulose synthase A catalytic subunit 6 [UDP-forming]	50,462,282	50,468,328
Leaf pub.	qLPA 2	Gh_A06G0019	PER64	Peroxidase 64	84,546	85,807
Leaf pub.	qLPA 2	Gh_A06G0018	EXPA1	Expansin-A1	82,222	83,190
Aphid rate	qAR 1	Gh_D09G0835	TPR1	Topless-related protein 1	32,915,885	32,923,301
Aphid rate	qAR 1	Gh_D09G0839	AGO5	Protein argonaute 5	33,188,202	33,202,318
Aphid rate	qAR 1	Gh_D09G0857	ZAT5	Zinc finger protein ZAT5	33,598,639	33,599,409
Aphid grade and Stem pub.	qAG 1 and qSPA 1	Gh_A06G1246	GLO4	Peroxisomal (S)-2-hydroxy-acid oxidase GLO4	89,813,435	89,822,109
Leaf pub.	qLPA 1	Gh_A06G1283	PDF2	Homeobox-leucine zipper protein PROTODERMAL FACTOR 2	92,578,328	92,581,427

Stem pub.: Stem Pubescence, Leaf pub.: Leaf Pubescence, AR: Aphid rate, AG: Aphid grade, SPA: Stem pubescence, LPA: leaf pubescence, bp: base pair.

## Supplementary Materials

Supplementary materials can be found at https://www.mdpi.com/2073-4425/11/4/368/s1. Figure S1: Physical and genetic linkage map; Figure S2: QTL Locations for plant response against Aphid; Table S1: Information about linkage map; Table S2: Comprehensive QTLs information; Table S3: Gene annotation for leaf and stem pubescence; Table S4: Gene annotation for response of plant against Aphid (Pests); Table S5: Primer sequence and gene IDs for qRT-PCR.
